# Toward an active exoskeleton with full energy autonomy

**DOI:** 10.3389/frobt.2025.1597271

**Published:** 2025-06-09

**Authors:** Yakir Knafo, Yinjie Zhou, Avi Manor, Alon Osovizky, Raziel Riemer

**Affiliations:** ^1^ Mechanical Engineering at Tel-Aviv University, Tel Aviv, Israel; ^2^ Electronics and Control Laboratories Nuclear Research Negev (NRCN), Beer-Sheva, Israel; ^3^ Industrial Engineering and Management Department of Ben-Gurion University of the Negev, Beer-Sheva, Israel; ^4^ Rotem Industries Ltd., Arava, Israel

**Keywords:** active exoskeleton, passive exoskeleton, harvesting energy, returning energy, regenerating mode, motoring mode

## Abstract

Exoskeletons aim to enhance human performance and reduce physical fatigue. However, one major challenge for active exoskeletons is the need for a power source. This demand is typically met with batteries, which limit the device’s operational time. This study presents a novel solution to this challenge: a design that enables the generation of electricity during motions where the muscles work as brakes and absorb energy, with the energy stored and subsequently returned to assist when the muscles function as motors. To achieve this goal, a knee exoskeleton design with a direct drive and a novel electronic board was designed and manufactured to capture the energy generated by the wearer’s movements and convert it into electrical energy. The harvested energy is stored in a power bank, and later, during motion, this energy is used to power the exoskeleton motor. Further, the device has torque control and can change the assistive profile and magnitude as needed for different assistance scenarios. Sit-to-stand (STS) motion was chosen as a test case for the first exoskeleton prototype. It was found that, during lowering (from stand to sit), the exoskeleton provided up to 10 Nm and harvested 9.4 J. During rising (from sit to stand), it provided up to 7.6 Nm and was able to return 6.8 J of the harvested energy. Therefore, the cycle efficiency of the exoskeleton system (return divided by harvesting) is 72.3%. In summary, this study introduces the first active exoskeleton for STS that can generate its own electrical power. The results show that the full development of this technology could reduce exoskeletons’ need for external energy sources.

## 1 Introduction

Exoskeletons are wearable robotic devices that augment human performance. They have a wide range of applications, including rehabilitation and industrial use. There are two main types of exoskeletons: passive devices, which rely on elements such as springs and clutches to aid the user’s movements and whose design is typically optimized to a single type of activity, such as running, walking, jumping (Nasiri et al., 2018; Collins et al., 2015; Ben-David et al., 2022), and active exoskeletons, which use motors to assist and can be used for several motions (Kim et al., 2019; Zhou et al., 2021). Active exoskeleton operation requires a reliable power source.

Traditional power sources, such as batteries and fuel cells, have a limited capacity, restricting the exoskeleton’s usable time. A novel approach to solving this problem could be a design that enables the generation of electricity during motions where the muscles work as brakes and return energy when the muscles act as motors (Riemer et al., 2021; Nuckols et al., 2020). Another definition for these phases is that the negative work phase is mechanical work at the joint level that is performed when angular velocity and torque are opposite in direction, whereas positive work is when both are working in the same direction (Winter 2009).

Biomechanical energy harvesters are devices that aim to generate electricity at a minimum or a reduction in effort by the user by targeting motions where the muscles work as brakes. A variety of biomechanical energy harvesters (Romero et al., 2009; Niu et al., 2004) have been developed that either generate electricity from the relative movement between limbs (Shepertycky and Li, 2015; Rubinshtein et al., 2012; Pozzi et al., 2012; Donelan et al., 2008; Chen, Chau, and Liao, 2017; Shepertycky et al., 2021; Fan et al., 2019; Cervera et al., 2016) or by the relative movement of a mass attached to the body (Martin and Li, 2019; Yuan and Zuo, 2016; Xie et al., 2017; Xie and Cai, 2015). Recent devices have shown that it is possible to harvest energy while reducing the metabolic effort (Gad et al., 2022; Shepertycky et al., 2021). Further, Shepertycky and colleagues were the first to show a device that reduces the effort by the user compared to walking with no device (Riemer et al., 2021; Shepertycky et al., 2021). All the above devices could only supply aid by replacing part of the muscle’s negative work during the motion, similar to regenerative braking in hybrid or electric cars. Another interesting study is a knee device that could harvest on a fixed resistor and use the generator as a motor, with a battery as its energy source (Shi et al., 2022). Most biomechanical energy harvesters can be viewed as exoskeletons that assist the joint during negative work and generate electricity. Where most harvesters focused on negative work performed at the knee joint during late swing phase in walking (e.g., Donelan et al., 2008; Shepertycky et al., 2021) Furthermore, to the best of our knowledge, there has been no active exoskeleton that can return the energy captured during the negative phase (e.g., late swing in walking or stand to sit) to assist motion during the positive phase (e.g., knee extension during mid stance or sit to stand). For an evaluation of existing harvesting devices, please see Supplementary Appendix SAI.

In standard operating mode, a brushless direct current (BLDC) motor operates in motoring (i.e., actuation) mode (Azam et al., 2013), converting electrical energy to produce mechanical energy. In a regenerative mode (Gurumurthy et al., 2013; Cervera et al., 2016), the BLDC actuator converts mechanical energy to electrical energy (Nian et al., 2014), which can then be stored in a battery or a power bank of supercapacitors. In contrasts with hybrid and electric vehicles (Long et al., 2013; Malode and Adware, 2016), where the motor turns in the same direction during both motoring and regenerative braking. In humans, the joint angular velocity typically features changes in direction and magnitude during one motion cycle (e.g., knee motion during walking) (Shepertycky et al., 2022). This creates a challenge in developing a single system that can perform torque and speed control for a motor/generator in a dual-operating mode, as these typically require two separate systems. There are off-the-shelf controllers (e.g., EPOS4, Maxon) designed to harvest energy automatically when detecting the negative power phase. However, it is impossible to set the harvesting torque profile in these controllers or modify the time in the motion when this harvesting occurs, which is crucial for harvesting only during muscles-negative work (Donelan et al., 2008; Shepertycky et al., 2021; Riemer and Shapiro, 2011).

Therefore, this study’s aim was to design, manufacture, and evaluate the first active exoskeleton with no external energy source. As a major building block, it is crucial to develop a torque control method based on a single BLDC (Mohammad and Khan, 2015; Kamalapathi et al., 2015) actuator that can seamlessly harvest and return energy to the user.

This research evaluates a lightweight prototype used to provide assistance during sit-to-stand assistance to able-bodied users or those with minor impairments. The methodology used for evaluations is similar to what was used in previous exoskeletons for sit-to-stand (Seko et al., 2019; Shepherd and Rouse, 2017). Sit-to-stand motion was chosen because they are important in human day-to-day functioning and could be lost due to stroke (Laschowski et al., 2019; Yoshioka et al., 2014) or other medical conditions. Furthermore, in the sit–stand motion cycle (stand–sit–stand), the knee joint performs negative work during the stand–sit phase (when the muscles are acting as brakes). Thus, the exoskeleton can provide assistance during the break and harvest energy in a manner similar to that of a hybrid car. During the sit–to–stand phase, the muscles perform positive work (acting as motors), while the exoskeleton returns energy by acting as a motor and thus reducing the work that the muscles are required to do. Thus, this motion represents a good case study with the potential for full energy autonomy and no need for an external power source.

## 2 Methods

### 2.1 System design and operation

Our new design is intended to enable harvesting and motoring using a single source of energy, which has never been done before. Thus, the design goal is to develop electronics and logic boards that will allow for the use of a single BLDC actuator as both a generator and motor for the energy-autonomous exoskeleton system. In addition, the system will include control of the torque profile and the timing of harvesting or returning energy. This section describes the system hardware design.

We used an exoskeleton with a direct drive to enable harvesting and motoring during knee flexion and extension. This exoskeleton was previously developed and tested in harvesting mode only during walking (Gad et al., 2022) For this exoskeleton, new specialized electronics and logic enabling a switch in operation mode (regenerative or motoring) were designed and manufactured. Further, a real-time control algorithm was implemented in a low-cost microcontroller.

#### 2.1.1 Mechanical system

**FIGURE 1:**
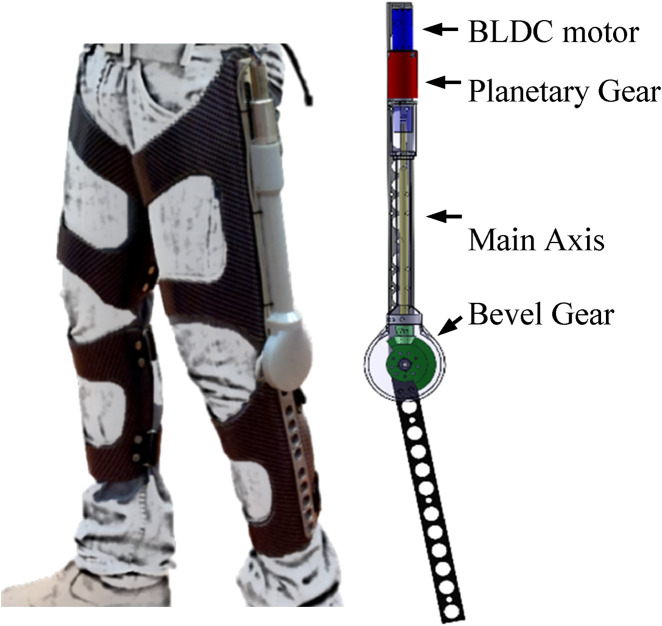
Mechanical design of the autonomous energy exoskeleton mounted on the knee brace (reproduced from Gad et al., 2022).

The apparatus of the harvesting and returning energy device is mounted on an orthopedic knee brace (Figure 1). The exoskeleton mechanism is based on a gear train that increases the slow angular velocity of the knee to the higher angular velocity required for the BLDC motor in harvesting and reduces the BLCD angular velocity to knee angular velocity in motoring.

The mechanical system consists of a customized knee brace, BLDC motor no. 323218 (EC-4pole 22 Ø22 mm) in direct drive connection, and a gear ratio of 243:1, with a 3:1 bevel gear and 81:1 planetary gear (measured weight of each device is approximately 1.5 kg) and mechanical system developed by Gad et al. (2022).

### 2.2 Electronic design of active control

**FIGURE 2:**
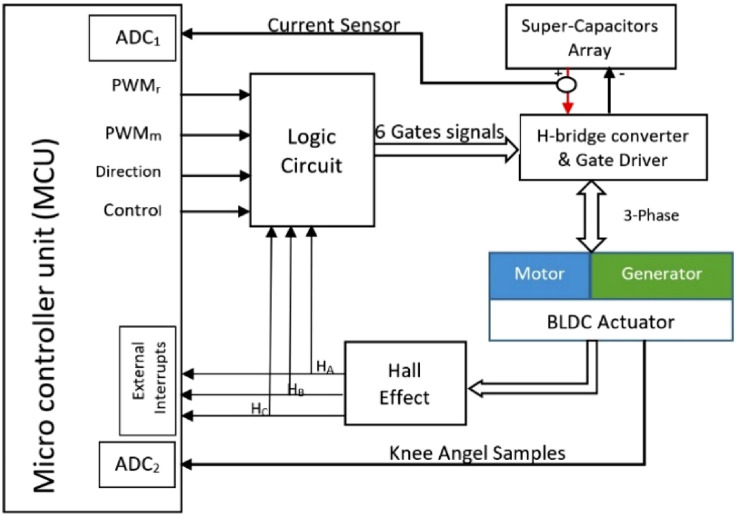
Block diagram of the active biomechanics control system, including the STM32 microcontroller, Hall sensors, BLDC motor, and logic circuit.

To enable the operation of the electrical motor both as a generator during harvesting energy and as a motor using a single power source, a new electronic system that includes the circuitry and electronic logic for torque control and power management was manufactured. The electronics (Figure 2) provide the control signals needed for active torque control in real-time by producing the correct electrical current profile for harvesting or driving the BLDC actuator.

Controlling the duty cycle of the pulse width modulation (PWM) (Mohammad and Khan, 2015; Kamalapathi et al., 2015) signals on active H-bridge transistors facilitate the direction and magnitude control of the current flowing through a BLDC actuator to a supercapacitor array and, subsequently, return the energy from the supercapacitor array to the BLDC actuator through the same active H-bridge when motoring mode is needed (motoring profile). By using PWM signals, the control system modulates the average current supplied to the motor, which in turn affects the motor’s speed and torque.

The microcontroller (STM32F103 cortex-M3 core device by STMicroelectronics) samples the knee joint angle (potentiometer) using an analog-to-digital converter (ADC) at 1 KHz. The knee angle is used to identify motion phases with positive and negative joint power and determine the BLDC motor operating mode (motoring or generating, the required direction of rotation, and the torque profile). The main idea behind the algorithm is that, during the stand–sit motion, the knee starts from approximately 0 (a straight leg) and is then flexed (an increasing angle). This is a signal for the harvesting to begin. In the sit–stand phase, the shifts move from approximately 90 to 0°; this means that the knee is in flexion, and the angle is decreasing (extension motion). Thus, it is a signal for the motoring to begin. The algorithm produces control and direction digital signals, and the PWM according to the desired current profile ${PWM}_{r}$ for the harvesting profile (regenerative mode) and ${PWM}_{m}$ for the desired returning energy (motoring mode) profile (see Supplementary Appendix SAII).

The logic circuit uses four control signals as inputs that are produced by the microcontroller and three outputs from the Hall Effect sensor (Ha, Hb, and Hc) and provides six switching signals (A_H_, A_L_, B_H_, B_L_, C_H,_ and C_L_) as inputs (see Supplementary Material S1) for the BLDC active commutation operation. The six switching signals are generated according to a set of Boolean equations (see Supplementary Material S2). The switching signals are amplified in a gate driver block driving an H-bridge metal-oxide-semiconductor field-effect transistor) MOSFET and, accordingly, controlling the BLDC actuator torque. The application of PWM signals on H-bridge transistors for harvesting and returning energy requires careful consideration of the circuit design. The H-bridge circuit must be designed to manage the voltage and current generated by the energy harvester.

The electronics power consumption is also a major consideration in a system with energy autonomy. The electronics include two modules: the microcontroller, with a current consumption of 20 mA, and the logic circuit, which consists of digital integrated circuits (IC) of high-speed CMOS logic gates that consume 300 uA. The two modules are fed by a voltage of 5 V, and the total power consumption is 101.5 mW. The energy source that feeds the electronic circuit is from the capacitors that store the harvested energy. This is a negligible consumption regarding the potential power production capacity of the biomechanical system. The total mass of the electronics and packaging is 0.5 kg.

### 2.3 System modeling and torque control

Since the exoskeleton should be able to provide different assistive torque profiles (both in shape and magnitude). The following design was implemented: the generator/motor is connected to the H-bridge, which is controlled by a microcontroller that generates the PWM signals according to the control law. The power bank is connected to the H-bridge power bus, allowing it to store the harvested energy or feed it to the motor.

**FIGURE 3:**
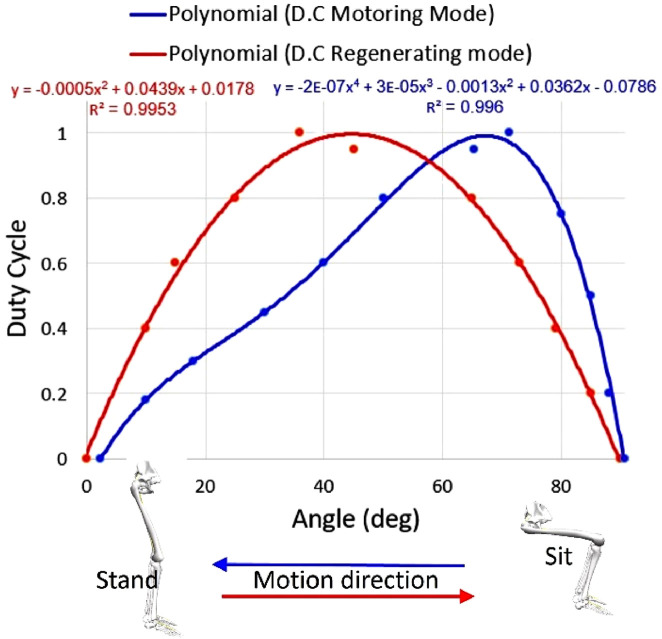
Changing the DC of PWM signals in regenerating mode during stand-to-sit motion (red) and motoring mode during sit-to-stand motion (blue).

To change the torque, the control law adjusts the duty cycle (DC) of the PWM signal. A higher duty cycle will result in a higher average current supplied to the motor, which would increase its torque. A lower-duty cycle will reduce the torque. The torque control was tested on an STS motion assistance task. In STS, power is harvested during the motion of going from a standing to a sitting position. While the user was moving from a sitting to a standing position, the harvested energy was used to drive the motor. The torque profile used to set the DC of the PWM signals is a polynomial of the 4th order or higher (Figure 3). These profiles were calculated using inverse dynamics (see the Experiment section) based on preliminary experiments with three participants who performed the sit-to-stand and stand-to-sit motions with and without the device (8–10 cycles each). These profiles were similar to those reported in previous literature (Laschowski et al., 2019; Yoshioka et al., 2014; Hurley et al., 2016).

Using the polynomial coefficient, it is possible to change the torque profile shape (timing of the peak and its height), for smooth control, PWM updates the DC every 1 ms (1000 Hz). To decrease the computational load in the microcontroller and ensure real-time operation, the DC value is obtained by using a lookup table that maps a set of input knee angle values to their corresponding output DC values. By pre-computing and storing a lookup table in the microcontroller memory, the calculation power required to generate the DC of the PWM signals is reduced.

#### 2.3.1 Torque model for the exoskeleton system

The applied torque on the knee is a combination of torque provided by the motor or generator, the device’s moment of inertia multiplied by angular acceleration, and the device’s mechanical friction, which also represents the electromechanical system efficiency (Equations 1, 2). Thus, the model for the regenerating and motoring modes of the exoskeleton device takes on the following form.

During harvesting (torque is against the direction of the rotation):

$$\tau_{knee-reg}=\left(\tau_{regenerating}\cdot N+\tau_{MOI}\right)\cdot \frac{1}{\eta }$$ (1)

During motoring (torque in the same direction as the rotation):

$$\tau_{knee-motor}=\left(\tau_{motoring}\cdot N+\tau_{MOI}\right)\cdot \eta$$ (2)

where $\tau_{knee-motor/reg}$ is the torque applied by the device at the knee, N is the gear ratio of the system, $\tau_{regenerating/motoring}$ is the torque applied by the BLDC actuator in the selected operation mode, $\tau_{MOI}$ is the torque due to the angular acceleration multiplied by the moment of inertia (MOI = 0.0327 kg · m^2^) of the system, and η=0.7 is the electromechanical efficiency of the system (Gad et al., 2022).

#### 2.3.2 Torque model for BLDC during regeneration

When the BLDC is in regeneration mode, the exoskeleton system will apply torque that resists the user’s motion. The model for the BLDC is derived using electrical analysis and the specific parameters of the system, such as the BLDC motor coefficient (see Supplementary Appendix SAIII) and the electronic design (Equation 3).

$$\tau_{regenerating}=\frac{K_{m}\cdot \left({\frac{\omega_{knee}\cdot N}{K_{V}}-V}_{Cap}\cdot {PWM}_{r}-{2\cdot V}_{DS-on}\right)}{\left(R_{p-p}+R_{wire}+R_{ESR}\right)}$$ (3)

where $K_{m}$ is the torque constant, $K_{V}$ is the speed constant of the BLDC motor, and $\omega_{knee}$ is the angular velocity of the knee. Multiplying $\omega_{knee}$ by G. R results in the BLDC motor angular velocity. $V_{Cap}$ is the voltage on the supercapacitor array at the operating point. ${PWM}_{r}$ is the control signal for the regenerating mode (a number between 0 and 1). $R_{p-p}$ is the terminal resistance of the motor between two phases. $R_{wire}$ is the total resistance of the wires. $R_{ESR}$ is the equivalent series resistance of the supercapacitor array. $\omega_{motor}$ is the BLDC motor angular velocity. $V_{DS-on}$ represents the MOSFET absolute maximum voltage between the drain and source.

#### 2.3.3 Torque model for BLDC during motoring

When the BLDC is in motoring mode, electrical energy is delivered back from the supercapacitor array to the motor in the same direction as the motion. Thus, the model of the BLDC applied torque has the following from (Equation 4):

$$\tau_{motoring}=\frac{K_{m}\cdot \left(V_{Cap}\cdot {PWM}_{m}-\frac{\omega_{knee}\cdot N}{K_{V}}-{2\cdot V}_{DS-on}\;\right)}{\left(R_{p-p}+R_{wire}+R_{ESR}\right)}$$ (4)

where ${PWM}_{m}$ is the control signal for the motoring mode.

### 2.4 Experiments

**FIGURE 4:**
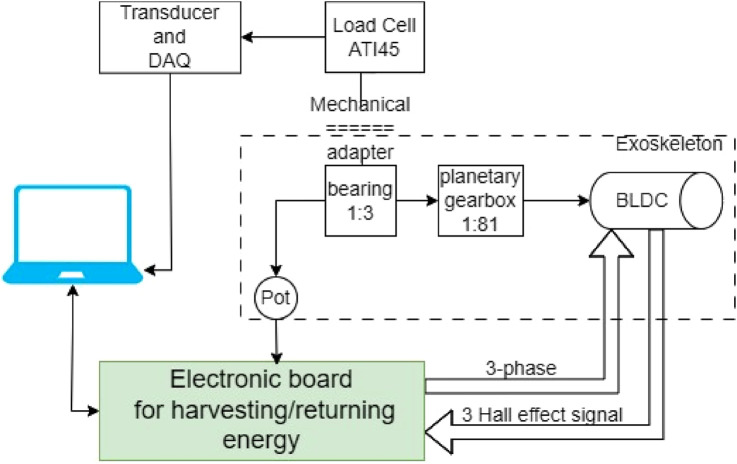
Block diagram describing the components used in the experimental setup. The computer is used to write to the controller and to read the load cell torque. The controller controls the assistive torque and its timing and profile.

The exoskeleton operation was evaluated using sit-to-stand motion. In a set of experiments, a healthy, 24-year-old male with a height of 180 cm and a weight of 76 kg was fitted with the two exoskeleton devices, one for each knee. These exoskeletons use the same mechanical design as our previous study (Gad et al., 2022; Figure 1), with a new electronic and control system that enables both harvesting and motoring. During the sit-to-stand trials, a load cell (mini-45, ATI, Apex, NC, USA) was used to measure the applied device’s torque on the knee joint (Figure 4). The seat height was 45 cm during all trials. Since the variability between and within subjects was found to be low in the sit-to-stand and stand-to-sit phases (Laschowski et al., 2019) we chose to use a single participant for this demonstration. The participant was instructed to perform 10 sit-to-stand cycles at approximately the same times for each phase, i.e., stand-to-sit motion for 1 s, then rest for 1 s in a seated position, and finally, sit-to-stand motion for 2 s. These times were based on the average self-paced times for performing these motions. To help maintain this timing, the motion cycle time was set to 4 s using a metronome that beeped every 2 s. The study was approved by the Ben-Gurion University of the Negev Human Research Institutional Review Board (1099-1).

**FIGURE 5:**
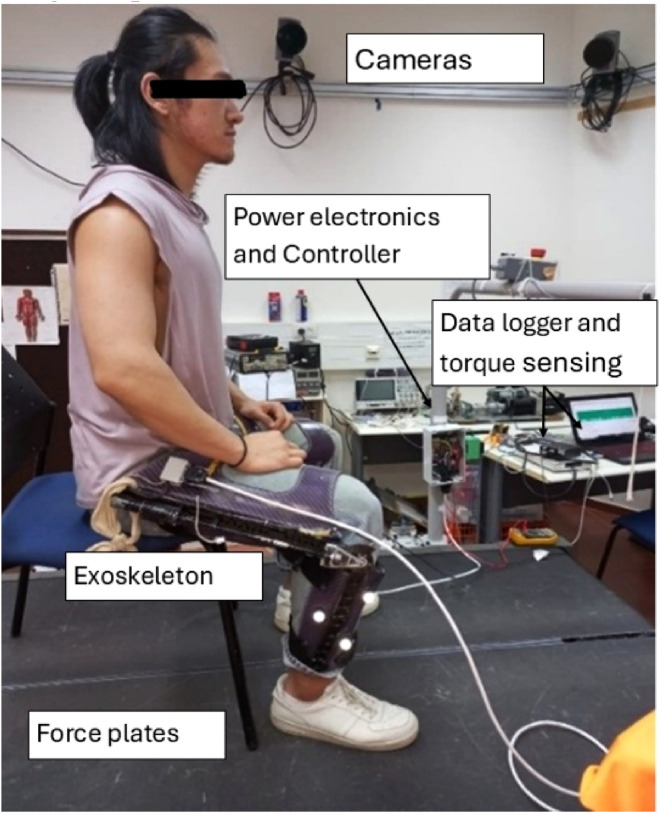
This figure shows the main experimental component in the lab.

During the experiment (Figure 5), the participant’s kinematics and kinetics were recorded using a 14-camera motion capture system (Qualisys, Gothenburg, Sweden) with a force plate (Bertec, Columbus, Ohio). Both the marker positions and ground reaction raw data were low-pass filtered (Butterworth second-order recursive), with a cut-off frequency of 10 Hz for marker positions and 20 Hz for GRF. The knee joint angle and torques were calculated using bottom-up method and 6DOF algorithm in visual 3D software (C-Motion). During the trials, the electronics on each leg were connected via USB connection to a laptop for data-logging, and the following data was registered: knee angle, knee torque magnitude and direction by the load cell sensor; a current sensor; the voltage level of the supercapacitor array; and the harvesting/returning control signal, which determines when to harvest/return and how much torque (based on the custom PWM profiles). The load cell sensor data were also filtered using low-pass filtering (Butterworth second-order recursive), with a cut-off frequency of 10 Hz.

The following experiments were performed to test the correct operation of the system:

First, to evaluate the accuracy of the torque model, its predictions were compared to the assistive torque values measured.

Second, tests were performed to verify the system’s ability to control the torque level (by PWM profile) in both motoring and harvesting modes. To test the ability to control the harvesting level in the stand-to-sit (lowering) trials, the ${PWM}_{r}$ control signal peak DC was set to three different levels—6.25% DC, 15% DC, and 30% DC—while maintaining the same profile shape. Where the level is defined as the set peak of the motor/generator divided by its maximum capability. For the ability to control the assistance in motoring (returning energy) mode in the sit-to-stand (rising) trials, the ${PWM}_{m}$ control signal peak levels were set to 0%, 50%, and 100% DC, demonstrating the ability to control the level of assistance via the torque profile. In the third experiment, the ability of the exoskeleton to change the assistive torque when motoring (returning energy) was tested using different control signals for returning energy at a 100% level (same peak, different timing). In addition to these experiments, the following calculations were made to determine the exoskeleton’s assistive torque (using the load cell as a torque meter) during both the stand-to-sit and sit-to-stand phases, and to represent how much energy was harvested (stored) and how much was returned. The total knee torque with an exoskeleton will be calculated using the bottom-up inverse dynamic method with the marker and force data. Where the difference between the total and the exoskeleton torque is the knee biological torque (Equation 5)

$$\tau_{biological}=\tau_{total}-\tau_{Exoskeleton}$$ (5)

All the experiment results presented represent an average of 8–10 cycles per experiment. During all the above tests, the supercapacitors were precharged to the nominal voltage of approximately 10 V. After that, they did not require any external energy to operate. Thus, the last test was a cyclic stand-to-sit and sit-to-stand test with a harvesting level of 30% duty cycle, with the purpose of measuring the number of cycles needed for self-charging the supercapacitor array to a working voltage level of approximately 10 V.

## 3 Experimental results

### 3.1 Evaluation of the torque model

**FIGURE 6:**
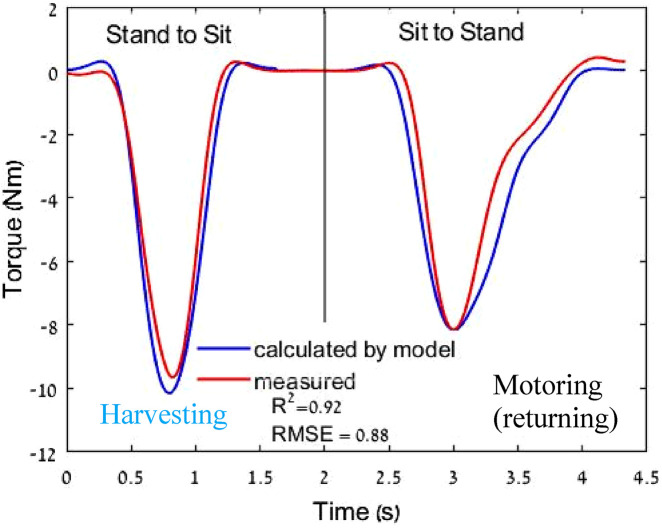
Example of torque applied to the knee in one sit-to-stand motion cycle and comparison between the load cell torque results and torque model. The first segment in the sit-to-stand motion is in harvesting mode, and the second segment is in returning mode.

The first experiment was conducted to evaluate the torque model’s accuracy. The load cell readings were compared with the harvesting/motoring profile predicted by the model. The torque measured by the model had a goodness of fit of R^2^ = 0.92 and RMSE = 0.88 (Figure 6).

### 3.2 Harvesting level effect on torque

**FIGURE 7:**
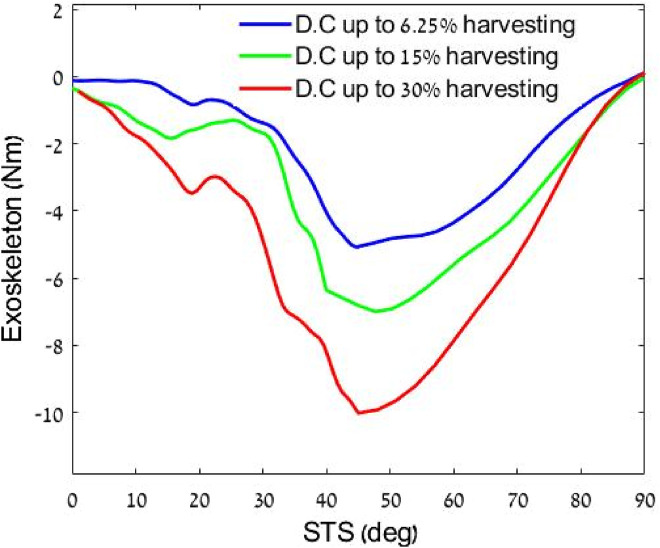
Effect of harvesting three levels on the torque profiles. All profiles are normalized by the knee angle. Where 0 is straight leg (standing) and 90° is bending when sitting.

The second experiment measured the harvesting energy for three (Figure 7) intensity profiles up to 6.25%, 15%, and 30% DC of the ${PWM}_{r}$ signal, resulting in torques 5.2 Nm, 7.6 Nm, and 10.3 Nm, respectively (during stand-to-sit motion where the knee joint is performing negative work).

### 3.3 Motoring level effect on torque

**FIGURE 8:**
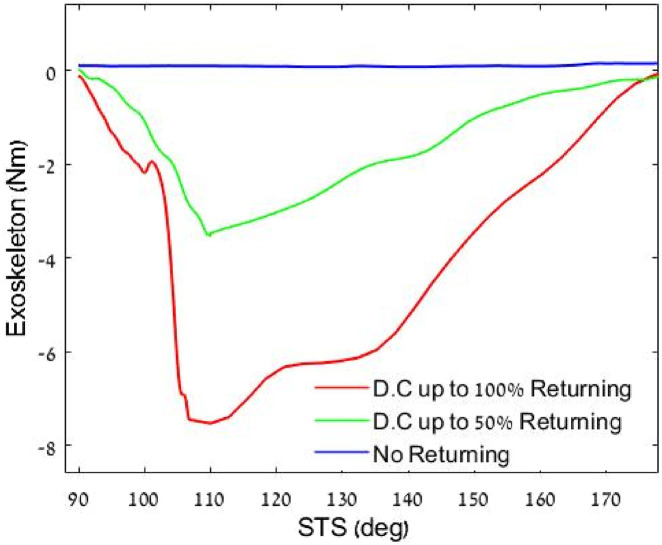
Effect of three levels of returning on the torque profiles, all profiles are normalized by the knee angle. Where 90° is bending when sitting, and 180 is straight leg (standing).

In the second experiment, we measured the torque of three motoring profiles in which 0%, 50%, and 100% DC peak values of the ${PWM}_{m}$ signal were used. The measured knee torques, which represent the energy return profiles during the sit-to-stand phase, achieved maximum torque values of 0 Nm, 3.6 Nm, and 7.6 Nm, respectively (positive work). In this experiment, harvesting was performed during stand-to-sit motion using a profile with a maximum of 30% DC (Figure 8).

**FIGURE 9:**
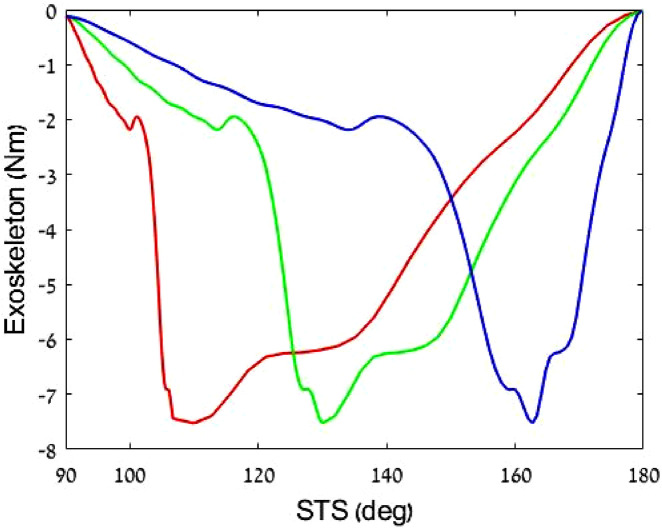
Three knee torque profiles demonstrate the system’s ability to change returning energy torque profiles. Red indicates higher torque at the beginning of the sit-to-stand motion, and blue represents higher assistive torque at the end of the motion.

In another test (Figure 9), different control signal profile shapes for returning energy were used to demonstrate the ability to control the assistance profile shape.

### 3.4 Demonstration of the exoskeleton’s ability to store energy and return

**FIGURE 10:**
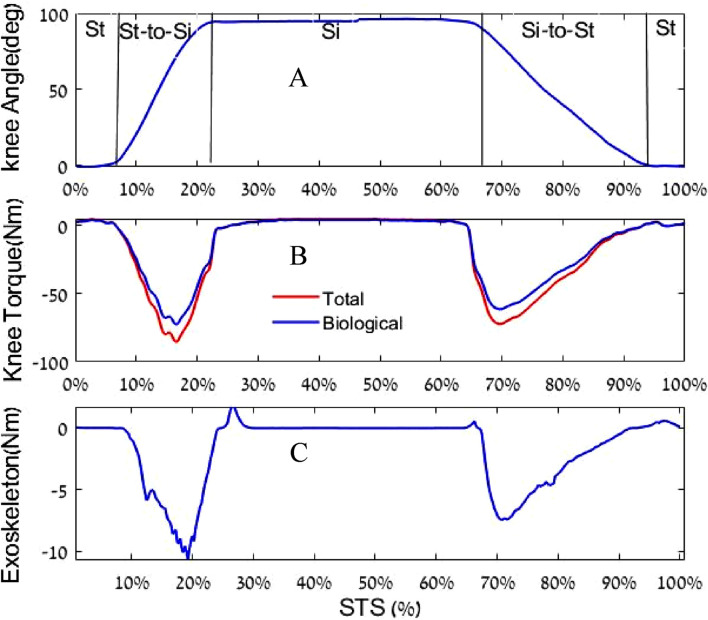
**(A)** Knee angle (zero is straight knee); **(B)** Knee torque (the blue curve refers to the biological knee torque with exoskeleton assistance, while the red curve refers to the total knee torque); and **(C)** Exoskeleton torque reaction. Note that “St” and “Si” denote stand and sit, respectively.

The results revealed that the total knee torque without an exoskeleton was 65–70 Nm. This torque was calculated using the bottom-up inverse dynamic method with the marker and force data. With our exoskeleton system, the maximum torque applied during the stand-to-sit motion was 10 Nm, while that during the sit-to-stand motion was 7.6 Nm (Figure 10).

The experiment’s results demonstrated that the proposed PWM active control method effectively harvests and returns harvested biomechanical energy during STS motion. The PWM controller was able to adjust the torque output of the exoskeleton in real time to match the torque demand of the wearer’s movement.

**FIGURE 11:**
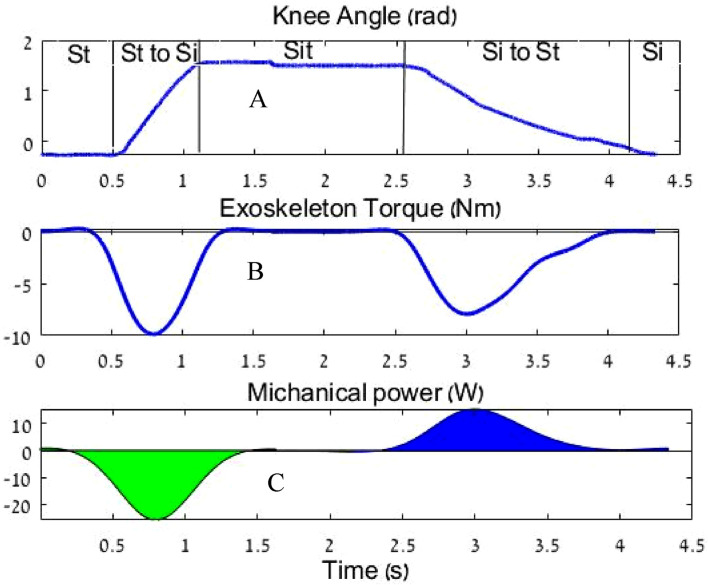
**(A)** Knee angle, **(B)** Exoskeleton torque, and **(C)** Mechanical power during sit-to-stand. The blue area is the assistance energy from the sit-to-stand motion; the green area is the harvested energy from the stand-to-sit motion.

To calculate the mechanical energy, an integration was performed on the mechanical power in the STS experiment (Figure 11). The harvested energy from standing-to-sitting motion was 9.4 J (which is approximately 16% of the total knee work), while the energy provided to assist the sitting-to-standing motion was 6.8 J (which is approximately 14% of the total knee work).

This calculation shows that the current system design allows for the supply of 72.3% of the mechanical harvested energy.

### 3.5 Self-charging

**FIGURE 12:**
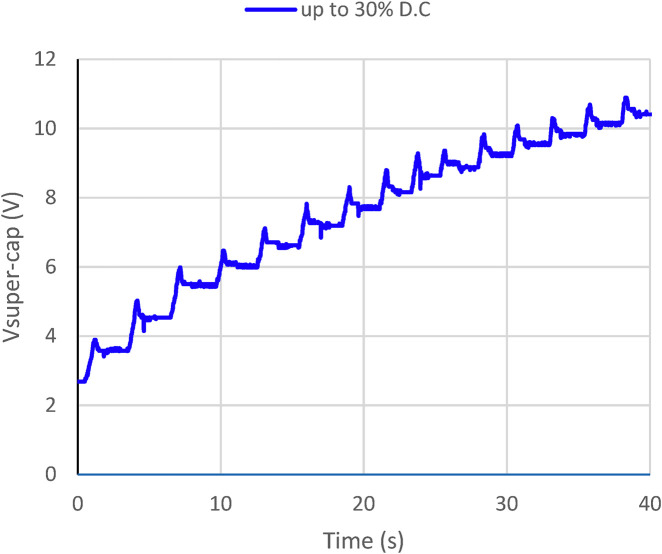
Supercapacitor array charging profile depends on the harvesting levels. The working voltage is approximately 10 V.

In the third experiment, we measured the supercapacitor array voltage in harvesting intensity profiles of 30% DC of the ${PWM}_{r}$ control signal. The power bank voltage level during this test is presented in Figure 12, where each local peak representing the harvesting part of a single STS cycle was found with a harvesting level of 30% DC. A working voltage of 10 V is achieved in about 12 sit-to-stand cycles. Another approach to reach an initial operating voltage level of 10 V is to pre-charge the supercapacitors once before use (after this first charge, the exoskeleton will not need additional energy). The initial charging of the supercapacitor array can take fewer cycles during this phase if the system will only perform harvesting.

## 4 Discussion

Currently, in the world of exoskeletons, low power consumption can only be achieved using passive or semi-passive devices, which are limited in their ability to assist a variety of motions. For example, they can assist only with level walking, not incline walking or stair climbing, as they are unable to change their assistance profile. For more flexibility in assisted motions, there is a need for active exoskeletons that typically have high energy requirements, which limit their operation time. In this study, for the first time, a prototype of a lightweight exoskeleton that can generate its own power, with the aim to aid able-bodied users or those with small impairments, is built and evaluated. To achieve this goal, a knee exoskeleton apparatus with a direct drive and a novel electronic board was designed and manufactured to capture the energy generated by the wearer’s movements and convert it into electrical energy. The harvested energy is stored in a power bank, and later, the motion is used to power the exoskeleton motor. As a first step, in the experiment with the single user, we evaluated and demonstrated the exoskeleton’s ability to aid the user in a stand-to-sit-to-stand motion.

In comparison to other devices designed for sit-to-stand assistance, the design of our exoskeleton applies no torque on the knee during sitting, regardless of the energy return profile. This is in contrast with previous devices, particularly the device developed by Seko et al. (2019), which is based on springs and applies the maximum torque during sitting, and that developed by Shepherd and Rouse (2017), which applies a smaller yet constant torque on the knee while sitting. This torque increases as the maximum torque profile increases and can reach up to 0.14 Nm/Kg during sitting (e.g., 11.2 Nm for an 80 Kg user). This means that in order for the user to sit with bent knees, he or she must apply a constant flexion torque to prevent the leg from being extended by the exoskeleton. However, in our device, there is no need for that, and the leg can rest. Further, because of the use of electronic control, our device exoskeleton has the ability to change and adjust the torque profile in both the energy harvesting and return modes by the PWM control signal in real-time without interfering with the mechanical design, something that is not possible in the spring-dominated device of Seko et al., (2019) or Shepherd and Rouse (2017) device.

## 5 Limitations and future work

The prototype exoskeleton used in the experiment was designed for walking and provides up to 15 Nm. This was a compromise between being lightweight and providing assistance that is relative to walking peek knee torques of approximately 50 Nm. Yet, for sit-to-stand movements with torques of 65–70 Nm, the designed exoskeleton provides only 6.7–10 Nm, and when we applied higher torque loads, the bevel gear emitted knocking sounds, indicating overstress or onset of failure mechanisms. Thus, future work should focus on the improvement of the mechanical structure. Furthermore, the working voltage level of the device’s supercapacitor, approximately 10 V, was set based on the self-determined pace of the sit-to-stand cycle. Thus, in this state—standing-to-sitting in the energy-harvesting mode—when the subject sits down quickly, more energy will be harvested, and any voltage created above the working point will break down on a load resistor.

However, in the sit-to-stand motion, which corresponds to the energy return mode, an increase in the angular velocity of the knee will result in a lower assistive torque. Further, if the knee angular velocity in the sit-to-stand phase is the same as the maximum rotational speed of the exoskeleton at its working point, the assistive torque will be zero.

A solution for both of these issues lies in the ability to shift the operating point to a voltage higher than 10 V. This will enable an increase in the assistive torque at the knee. For example, assuming the voltage at the operating point will be 28 V, according to the motoring model (4), the assistance to the knee will be approximately 21.3 Nm. This change in working voltage could be achieved using a new design with a variable-voltage control system that dynamically adjusts the operating point based on real-time feedback from the user’s motion. This system could be implemented using electronic components, such as DC-DC converters (e.g., buck-boost converters), to regulate the voltage and microcontroller, combined with adaptive control algorithms and angular sensors (which the exoskeleton already has) to provide real-time feedback. It should be noted that this prototype aims to assist users with some ability to perform the motion. For the elderly population or people with minor disabilities, even a small amount of help may be needed to complete the task. Even at its current state, our device provides approximately 10% of the capability of a healthy knee during sit-to-stand motion. Last, in this paper, we only focused on the development of the electronic system and showed the ability to control torque. Future research should improve the mechanical design and advanced control algorithms (e.g., (Wang et al., 2023)) to enable assistance with movements such as walking and running, as well as measure the effect on metabolic power.

## 6 Conclusion

This paper presents an exoskeleton with the ability to harvest and return energy with an adjustable profile for the sit-to-stand motion. Returning harvested energy has the potential to significantly improve the practicality of exoskeletons in various applications, including medical rehabilitation, industrial work, and military use, by reducing the need for frequent battery replacements or external power sources.

The presented exoskeleton system is presented to be energy-autonomous in the STS scenario. It represents an important step forward in the evolution of exoskeleton technology, and we can expect to see further advancements in this field in the coming years.

## Data Availability

The original contributions presented in the study are included in the article/Supplementary Material, further inquiries can be directed to the corresponding author.
