# Supplementary material for: Toward an active exoskeleton with full energy autonomy
Source: Front Robot AI. 2025 Jun 9;12:1597271. doi: 10.3389/frobt.2025.1597271 (PMC12183371; doi:10.3389/frobt.2025.1597271)
Supplement: Supplementary file 1 [file Supplementaryfile1.docx]

Supplementary Material

# Signals Connection

In a BLDC motor, the stator windings are arranged in a specific pattern, typically 3-phase, and are energized in a sequence to create a rotating magnetic field. This rotating magnetic field interacts with the permanent magnets in the rotor to produce mechanical rotation.

The developed logic circuit integrates PWM's control signals with switching signals and creates switching patterns. The switching patterns are represented in terms of Boolean equations.

The logic circuit needs to support operating the BLDC motor in energy regenerative mode and motoring mode. This dual-mode operation needs to have a two-way clockwise (CW) and counterclockwise (CCW) rotation.


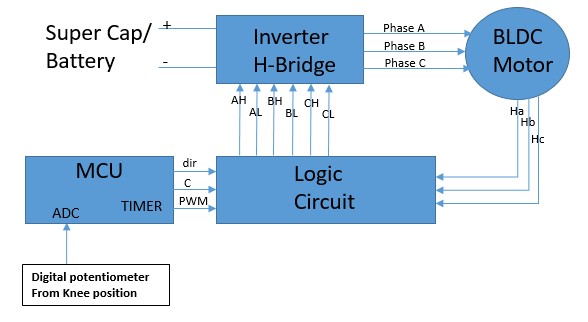


Supplementary Figure 1. Signals Connection Between the Sub-Units on the Electronic Board

# Motoring Mode Commutation

The states of switching control signals for implementing the logic necessary to operate the BLDC in dual mode: Motoring mode (Supplementary Table 2.1) and Regenerating mode (Supplementary Table 2.2).

## Motoring Mode Commutation

The motoring mode in a two-way (CW and CCW) rotation state

| Interval | Direction  (D) | Hall sensors | | | AH | AL | BH | BL | CH | CL |
| --- | --- | --- | --- | --- | --- | --- | --- | --- | --- | --- |
|  |  | HA | HB | HC |  |  |  |  |  |  |
| I | 1 | 1 | 0 | 1 | 0 | 0 | 0 | 1 | 1 | 0 |
| II |  | 1 | 0 | 0 | 1 | 0 | 0 | 1 | 0 | 0 |
| III |  | 1 | 1 | 0 | 1 | 0 | 0 | 0 | 0 | 1 |
| IV |  | 0 | 1 | 0 | 0 | 0 | 1 | 0 | 0 | 1 |
| V |  | 0 | 1 | 1 | 0 | 1 | 1 | 0 | 0 | 0 |
| VI |  | 0 | 0 | 1 | 0 | 1 | 0 | 0 | 1 | 0 |
| I | 0 | 1 | 0 | 1 | 0 | 0 | 1 | 0 | 0 | 1 |
| II |  | 1 | 0 | 0 | 0 | 1 | 1 | 0 | 0 | 0 |
| III |  | 1 | 1 | 0 | 0 | 1 | 0 | 0 | 1 | 0 |
| IV |  | 0 | 1 | 0 | 0 | 0 | 0 | 1 | 1 | 0 |
| V |  | 0 | 1 | 1 | 1 | 0 | 0 | 1 | 0 | 0 |
| VI |  | 0 | 0 | 1 | 1 | 0 | 0 | 0 | 0 | 1 |

Supplementary Table 2. 1 Boolean table for Motoring mode

From Supplementary Table 2.1, we create these six Boolean equations (1)-(6)

$A_{H}={(D\cdot H}_{A}\cdot\bar{H_{C}}+\bar{D}\cdot\bar{H_{A}}\cdot H_{C})\cdot{PWM}_{m}$ (1)

$A_{L}=\left( D\cdot\bar{H_{A}}\cdot H_{C}+\bar{D}\cdot H_{A}\cdot\bar{H_{C}} \right)$ (2)

$B_{H}=(D\cdot\bar{H_{A}}\cdot H_{B}+\bar{D}\cdot H_{A}\cdot\bar{H_{B}})\cdot{PWM}_{m}$ (3)

$B_{L}=(D\cdot H_{A}\cdot\bar{H_{B}}+\bar{D}\cdot\bar{H_{A}}\cdot H_{B})$ (4)

$C_{H}=(D\cdot\bar{H_{B}}\cdot H_{C}+\bar{D}\cdot H_{B}\cdot\bar{H_{C}})\cdot{PWM}_{m}$ (5)

$C_{L}=(D\cdot H_{B}\cdot\bar{H_{C}}+\bar{D}\cdot\bar{H_{B}}\cdot H_{C})$ (6)

where ${PWM}_{m}$ is the pulse width modulation signal for motoring mode and D is the indicate direction rotation signal.

## Regenerating Mode Commutation

The regenerating mode in two-way (CW and CCW) rotation State

| Interval | Direction  (D) | Hall sensors | | | AH | AL | BH | BL | CH | CL |
| --- | --- | --- | --- | --- | --- | --- | --- | --- | --- | --- |
|  |  | HA | HB | HC |  |  |  |  |  |  |
| I | 1 | 1 | 0 | 1 | 0 | 0 | 1 | 0 | 0 | 1 |
| II |  | 1 | 0 | 0 | 0 | 1 | 1 | 0 | 0 | 0 |
| III |  | 1 | 1 | 0 | 0 | 1 | 0 | 0 | 1 | 0 |
| IV |  | 0 | 1 | 0 | 0 | 0 | 0 | 1 | 1 | 0 |
| V |  | 0 | 1 | 1 | 1 | 0 | 0 | 1 | 0 | 0 |
| VI |  | 0 | 0 | 1 | 1 | 0 | 0 | 0 | 0 | 1 |
| I | 0 | 1 | 0 | 1 | 0 | 0 | 0 | 1 | 1 | 0 |
| II |  | 1 | 0 | 0 | 1 | 0 | 0 | 1 | 0 | 0 |
| III |  | 1 | 1 | 0 | 1 | 0 | 0 | 0 | 0 | 1 |
| IV |  | 0 | 1 | 0 | 0 | 0 | 1 | 0 | 0 | 1 |
| V |  | 0 | 1 | 1 | 0 | 1 | 1 | 0 | 0 | 0 |
| VI |  | 0 | 0 | 1 | 0 | 1 | 0 | 0 | 1 | 0 |

Supplementary Table S2. 2 Boolean table for Motoring mode

From Supplementary Table 2.2, we create these six Boolean equations (7)-(12)

$A_{H}={(D\cdot\bar{H_{A}} \cdot H}_{C}+\bar{D}\cdot H_{A}\cdot\bar{H_{C}})\cdot{PWM}_{r}$ (7)

$A_{L}=\left( D\cdot H_{A}\cdot\bar{H_{C}}+\bar{D}\cdot\bar{H_{A}}\cdot H_{C} \right)\cdot{PWM}_{r}$ (8)

$B_{H}=(D\cdot H_{A}\cdot\bar{H_{B}}+\bar{D}\cdot\bar{H_{A}}\cdot H_{B})\cdot{PWM}_{r}$ (9)

$B_{L}=(D\cdot\bar{H_{A}}\cdot H_{B}+\bar{D}\cdot H_{A}\cdot\bar{H_{B}})\cdot{PWM}_{r}$ (10)

$C_{H}=(D\cdot H_{B}\cdot\bar{H_{C}}+\bar{D}\cdot\bar{H_{B}}\cdot H_{C})\cdot{PWM}_{r}$ (11)

$C_{L}=(D\cdot\bar{H_{B}}\cdot H_{C}+\bar{D}\cdot H_{B}\cdot\bar{H_{C}})\cdot{PWM}_{r}$ (12)

where ${PWM}_{r}$ is the pulse width modulation signal for regenerative braking modes.

## Dual Mode Operation

The equations are developed for separate cases. Equations (1)–(6) represent the motoring mode of operation, and equations (7)–(12) represent the regenerative mode of operation. These operation modes can be combined using one control signal.

| C (Control Signal) | BLDC Operation Mode |
| --- | --- |
| "1" | Motoring |
| "0" | Regenerative braking |

Supplementary Table 2. 3 Control signal state

Table IV introduces a control signal to combine both analyses. The motoring mode takes place when C is high state “1”, and regenerative braking takes place when C is low state “0”. Now, (1)–(6) and (7)–(12) can be combined by adding the control signal.

Finally, the combined Boolean equations based on (1)–(6) and (7)–(12) that include direction and control signals can be formed using the OR operator.

$A_{H}={{C\cdot(D\cdot H}_{A}\cdot\bar{H_{C}}+\bar{D}\cdot\bar{H_{A}}\cdot H_{C})\cdot{PWM}_{m}+\bar{C}\cdot(D\cdot\bar{H_{A}} \cdot H}_{C}+\bar{D}\cdot H_{A}\cdot\bar{H_{C}})\cdot{PWM}_{r}$ *(13)*

$A_{L}=C\cdot\left( D\cdot\bar{H_{A}}\cdot H_{C}+\bar{D}\cdot H_{A}\cdot\bar{H_{C}} \right)+\bar{C}\cdot\left( D\cdot H_{A}\cdot\bar{H_{C}}+\bar{D}\cdot\bar{H_{A}}\cdot H_{C} \right)\cdot{PWM}_{r}$ *(14)*

$B_{H}=C\cdot\left( D\cdot\bar{H_{A}}\cdot H_{B}+\bar{D}\cdot H_{A}\cdot\bar{H_{B}} \right)\cdot{PWM}_{m}+\bar{C}\cdot(D\cdot H_{A}\cdot\bar{H_{B}}+\bar{D}\cdot\bar{H_{A}}\cdot H_{B})\cdot{PWM}_{r}$ *(15)*

$B_{L}=C\cdot\left( D\cdot H_{A}\cdot\bar{H_{B}}+\bar{D}\cdot\bar{H_{A}}\cdot H_{B} \right)+\bar{C}\cdot(D\cdot\bar{H_{A}}\cdot H_{B}+\bar{D}\cdot H_{A}\cdot\bar{H_{B}})\cdot{PWM}_{r}$ *(16)*

$C_{H}=C\cdot\left( D\cdot\bar{H_{B}}\cdot H_{C}+\bar{D}\cdot H_{B}\cdot\bar{H_{C}} \right)\cdot{PWM}_{m}+\bar{C}\cdot(D\cdot H_{B}\cdot\bar{H_{C}}+\bar{D}\cdot\bar{H_{B}}\cdot H_{C})\cdot{PWM}_{r}$ *(17)*

$C_{L}=C\cdot\left( D\cdot H_{B}\cdot\bar{H_{C}}+\bar{D}\cdot\bar{H_{B}}\cdot H_{C} \right)+\bar{C}(D\cdot\bar{H_{B}}\cdot H_{C}+\bar{D}\cdot H_{B}\cdot\bar{H_{C}})\cdot{PWM}_{r}$ *(18)*

To minimize the hardware of the six Boolean equations, we can see common Boolean expressions in (13) and (14), (15) and (16), and (17) and (18). First, the common Boolean expressions need to be created and then reused in the other equation.

Appendix

# Appendix I: Comparison of the capabilities of different Exoskeleton devices

| Article | Return Energy | One/Two Direction | Single/Dual Source |
| --- | --- | --- | --- |
| Pozzi et al. (Pozzi et al. 2012) | 🗶 | One | -- |
| Donelan et al. (Donelan et al. 2008) | 🗶 | One | -- |
| Chen et al. (Chen, Chau, and Liao 2017) | 🗶 | Two | -- |
| Shepertcky et al. (Shepertycky et al. 2021) | 🗶 | One | -- |
| Fan et al. (Fan et al. 2019) | 🗶 | One | -- |
| Martin et al. (Martin and Li 2019) | 🗶 | Two | -- |
| Cervara et al. (Cervera et al. 2016), Gad et al. (Gad et al. 2022) | 🗶 | Two | -- |
| Shi et al. (Shi et al. 2022) | ✓ | One | Dual |
| Our study | ✓ | Two | Single |

Description of the columns:

- Return Energy indicates whether the exoskeletons can return energy by operating in motoring mode and giving assistance to the users.
- One/Two Directions indicate whether the exoskeleton can harvest or return energy clockwise (cw) and counterclockwise (ccw) of the knee motion.
- Single/Dual Source indicates whether the exoskeleton performs energy return from the same energy source or from a separate energy source.

# Appendix II: Developed Electronic Board on Microcontroller STM32F103C8T6
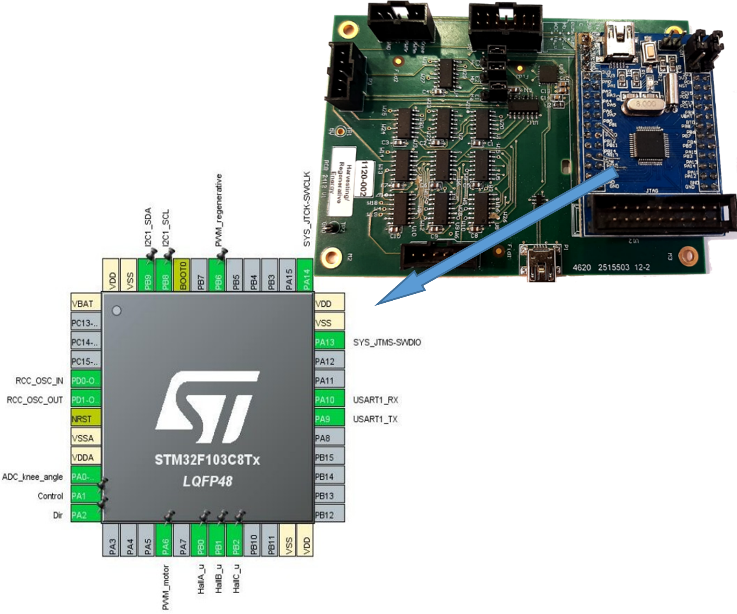


Fig. 13. Electronic board and microcontroller pinout.

TABLE Ⅰ: Microcontroller Pin Configuration

| Pin Number | - Pin Name (function) | - Alternate Function(s) | - Label |
| --- | --- | --- | --- |
| 5, 6 | PD0-OSC_IN/OUT |  | System clock |
| 10 | PA0 | ADC1_IN0 | ADC_knee_angle |
| 11 | PA1 | GPIO_Output | Control |
| 12 | PA2 | GPIO_Output | Dir |
| 16 | PA6 | TIM3_CH1 | PWMm_motoring |
| 18 | PB0 | GPIO_EXTI0 | HallA_u |
| 19 | PB1 | GPIO_EXTI1 | HallB_u |
| 20 | PB2 | GPIO_EXTI2 | HallC_u |
| 30 | PA9 | USART1_TX | Communication _Tx |
| 31 | PA10 | USART1_RX | Communication _Rx |
| 34 | PA13 | SYS_JTMS-SWDIO | Debug uses |
| 42 | PB6 | TIM4_CH1 | PWMr_regenerative |

# Appendix III: BLDC EC-4POLE 22 323218 Coefficients

| Motor Data | Coefficients |
| --- | --- |
| Max. efficiency | 89 (%) |
| Terminal resistance phase to phase | 0.527 (Ω) |
| Torque constant | 14 (mNm/A) |
| Speed constant | 680 (rpm/V) |
| Rotor inertia | 5.54 (gcm^2^) |
